# How the Labor Market Affects the Self-Perceived Health of Older Workers. The Evidence From Central and Eastern European Countries (CEECs)

**DOI:** 10.3389/fpubh.2021.655859

**Published:** 2021-07-05

**Authors:** Paulina Ucieklak-Jeż, Agnieszka Bem

**Affiliations:** ^1^Faculty of Law and Economics, Jan Długosz University, Czestochowa, Poland; ^2^Department of Corporate and Public Finance, Wrocław University of Economics and Business, Wrocław, Poland

**Keywords:** social determinants of health, self-perceived health, key indicators of the labor market, unemployment, inactivity

## Abstract

**Background:** The paper aims to analyze the impact of key labor market indicators on the self-assessed health of the population of older workers (aged 55–64).

**Methods:** Authors build the econometric models where the dependent variable is the self-perceived health status (for women and men separately). Explanatory variables are selected key indicators of the labor market, covering unemployment, including long-term, inactivity, or under-employment. The average household income is used to control the effect of wealth. Additionally, the models incorporate the variable describing the proximity of retirement. The research sample consists of nine countries of Central and Eastern Europe: Poland, Czech Republic, Slovakia, Hungary, Lithuania, Latvia, Estonia, Bulgaria, and Romania.

**Results and Conclusions:** The study confirms that in the group of elderly workers, the perceived state of health is influenced by long-term unemployment, inactivity, and, in the case of women, time-related underemployment.

## Introduction

Rich literature describes how the social gradient affects the population's health. Social determinants of health (SDH) consist of non-medical factors deriving from the social and economic environment—these dimensions significantly affect the health state (1). The most commonly noted socio-economic factors shaping health are income, social status, social support network, education and literacy, physical environment, environmental quality. The list is still open due to the complexity of a phenomenon of health (2).

Among the determinants listed above, employment status and working conditions are perceived as crucial. As a result, several studies of SDH include more or fewer variables related to the labor market. These are, for example, the employment status (3) or occupation (4–6). From the first look, a link between good health and unemployment seems to be intuitive. There is a general assumption that employment and healthful working conditions promote good physical and mental health. However, previous studies provide inconsistent results, and the debate in this area is still open.

Importantly for further analysis, the link between health and labor market status is bidirectional. Better health promotes employment and increases income (7). Being unemployed contributes to higher mortality (8) or deteriorates the perceived health (9). As summarized by Maarten and Marcel, health and work are endogenously related because of a direct causal impact of health on work and vice versa (9).

The study aims to analyze the impact of selected labor market indicators on self-assessed health. It gives a fresh look at the decomposed problem of unemployment, including long-term unemployment or labor market inactivity, based on critical indicators of the labor market (KILM) developed by the International Labor Organization. The research focuses on the age group 55–64; this group at higher risk of unemployment or permanent inactivity. Along with age, the health status deteriorates—it is reflected both in mortality rates and subjective health state self-assessment. Additionally, the decisions of elderly workers related to their professional activity can be affected by social, cultural, and economic factors and perceived health. The choice of this age group also responds to the current demographic problems—the process of aging societies, manifested by the declining share of young workers, encourages employers to retain their employees representing older age groups. It can be one of the possible answers to the demographic problems, but only if they are ready to extend their working lives.

The research sample covers nine countries of Central and Eastern Europe (CEE). Although their demographic situation is currently relatively favorable compared to western European countries, this could change drastically in the coming decades. The fall of communism and the economic transition has dramatically declined fertility rates, which will soon affect the labor market (10). Demographic challenges, which are already apparent in developed countries of West Europe, will stoke the CEE countries, with increased force, in the perspective of 20 years.

## Literature Context

As previously mentioned, the labor market's impact on health status is still a vital research area. It is mainly due to previous inconsistent results. The reasons for this “inconsistency” are multiple. Most of all, the labor market is a comprehensive concept and can be analyzed from different perspectives. Finally, the researchers usually focus on two main areas: working conditions (including social factors) or a broader picture of unemployment, including losing a job, long-term unemployment, or inactivity. Different measures of health state (mortalities, morbidities, life expectancies, or self-assessment) are the next potential source of conflicting results.

Generally, the previous findings, in the area of the relationship between unemployment and health, can be split into two categories—studies that support the hypothesis of the detrimental effect of unemployment on health, mainly via a mechanism of lowered income and impaired social status, and results that suggest quite the opposite mechanism.

Many works confirm that a loss of a job, or broadly being unemployed, impacts health negatively. Brenner was the first who described this inverse relationship. Therefore, in the literature, it is often called the “Brenner hypothesis” (11, 12). This undesirable effect is mainly rooted in the loss or reduction of income, which results from lowered economic activity, sometimes leading to poverty. This situation may worsen self-persisted health individually, but it is often combined with other unfavorable factors related to the social environment (13).

Inspired by Preston's law, researchers usually accept the assumption that higher-income individuals are healthier than those with lower incomes. Several previous studies confirm this thesis (10), often demonstrated by higher mortality rates after a job loss (14) and significantly evident in the men subpopulation (14, 15). Unemployment may also sharply decrease subjective well-being (16). This effect is more apparent when the time unemployment extends to long-term one (17–20). The unemployed individuals also carry a higher burden of diseases than those who work, even if it is only a part-time job (21).

One of the recent studies by Economou et al. (22), based on European countries' data, supports the view that unemployment harms health—when the unemployment increases by 1%, the mortality rises approximately per 1.54 deaths for every 100,000 inhabitants. This finding is exciting as authors tend to control the confounding factors in the analyzed relationship. It is also in line with Crost and Friedson (23). They estimate that a 1% increase in the group-specific unemployment rate is associated with an ~0.015% increase in the group-specific mortality rate. Additionally, Catalano et al. (24), job loss increases the risk of premature mortality, and, according to Eliason and Storrie (14), such experience rises by 44% of men's risk of death.

This relationship between job status and health has potentially many explanations. As a result of job loss, lowered income usually translates into lower availability of health services, especially in private insurance systems. Having or not having health insurance is, in several countries, strongly linked to the labor market. As confirmed by Van Doorslaer et al. (25), people with higher incomes are significantly more likely to benefit from medical consultations than people with lower incomes (26). However, the consequences of unemployment go far beyond a simple reduction of income. Several studies emphasize additional psychological factors like the stigma and isolation related to unemployment (27). This situation is comparable with the negative consequences of retirement (28), linked to lowered income and broken social networks (29).

The inverse relationship was pioneered by the works of Ruhm (30–32), Gerdtham and Ruhm (33), Neumayer (34), and Tapia Granados (35). Gerdtham and Ruhm (33) confirm that a 0.4% growth of mortality accompanies the 1% decrease in the unemployment rate. This negative relationship is confirmed by several further studies, among all by Ariizumi and Schirle (36) for middle-aged Canadians. Birgisdóttir and Ásgeirsdóttir (37) report the pro-cyclical nature of unemployment for the middle-aged Iceland population. Tapia Granados and Ionides (38) estimate that when unemployment increases by 1%, it is linked to mortality drop by 0.5%. Haaland and Telle (39) fortify this result by observing that other factors related to deteriorated health also have a pro-cyclical character. The impact of low education and poor health on unemployment varies by the work-life stage (40). Deficient health levels are observed for residents of underdeveloped areas and those at risk of poverty or unemployment (41). According to the European Commission, higher morbidity, and premature mortality rates are observed in groups of illegally employed, homeless, and single parents (42).

To summarize, earlier studies usually suggest that unemployment affects a population's health negatively. It seems that, although the evidence supporting this hypothesis is relatively new, the latest studies suggest quite remote conclusions. Tapia Granados and Ionides partially described this problem, and they found that in the second half of the twentieth century, economic growth started to affect health negatively, in contrast to earlier decades (38).

Some differences in previous results may also be rooted in analyzed countries' characteristics, as Hessel and colleagues report (43). Contextual factors, including policy responses, may have contributed to the different results. Health inequalities, by employment status, increase significantly by 72% in men and 16% in women after controlling covariates. Those trends are partly mediated by consequences of unemployment, such as income loss, income poverty, life satisfaction, and economic sorrows. Using regression models for panel data, the authors confirm that the observed increases in health inequalities at the population level also exist at the level of individuals (44).

## Materials and Methods

### Source of Data

Data covering the years 2005–2018 for nine countries of Central and Eastern Europe (CEE): Poland (PL), Czech Republic (CR), Slovakia (SL), Hungary (HUN), Lithuania (LT), Latvia (LV), Estonia (EST), Bulgaria (BUL), and Romania (ROM) are investigated. This research group is consistent in terms of economic development, demography, culture, or historical baggage despite their different population potential. The data come from the following sources: EUROSTAT and LABORSTA, provided by the International Labor Organization (ILO).

### Model

The very formulation of the hypothesis on the health effects of unemployment appears to be risky in the context of previous studies; hence the research bases mainly on exploratory data analysis, focusing on the effects of individual labor market characteristics on the health of older workers. Several aspects of the labor market are considered, like unemployment, including long-term and time-related underemployment, labor inactivity, and informal economy employment.

The following research questions are formulated:

1. Which characteristics of the labor market influence health status more significantly?
2. Is the strength and direction of this relationship the same for men and women subpopulations?

To answer those questions, two GMM models, separately for men and women, are calculated (45, 46). GMM models help avoid OLS method requirements, usually challenging to fulfill, like the normality of variable distributions. GMM allows estimating the non-linear parameters of the dynamic panel models. Additionally, GMM is more robust than other methods of estimation (47, 48). Such an active panel model help to address the endogeneity problem caused by reverse causality between health and its social determinants (49).

In the model based on the first differences, there are no personal effects. The assumption that there is no correlation between explanatory variables and particular outcome results is no longer required. The use of instrumental variables eliminates the problem of endogeneity of variables and estimator mismatch (50).

The Chow test examines the stability of the model parameters.

### Dependent Variables

The dependent variable describes the populations' health state (variable SP18). Generally, there are two types of health state measures—objective and subjective. The most popular objective measures are mortalities or life expectancies. They are reliable as they base on actual events and public statistics. The subjective indicators require survey research where respondents are asked to assess their health state (both physical and mental), usually using the five-degree Likert scale (51).

The subjective indicators, especially self-assessed health (SAH), although biased by their subjective component, are very inclusive and capture all aspects of health (both mental and physical, including functional and well-being dimensions) not covered by other health variables (52–57). Hence, SAH is a strong predictor of mortality (52, 57, 58).

Apart from this strong advantage, SAH is also biased by socio-economic factors like gender, age, income, occupation, race, cultural background (57–61). Different age groups are governed by different factors (61, 62). Middle-aged respondents assess their health through the prism of symptoms and psychic well-being, while the elderly rather face chronic diseases (63). There are also differences rooted in gender—some studies report that SAH is a better predictor of men's mortality (64). Countries heterogeneities may also affect the results—Southern and Central and Eastern Europeans are much more likely to misreport their physical and cognitive abilities than Northern and Western Europeans.

This research bases on SAH for the population aged 55–64. The data concerning SAH comes from the European health interview survey (EHIS). Respondents assess the general perceived health by answering the question: “How is your health in general? Is it …” Very good/Good/Fair/Bad/Very bad? It is a standardized question recommended by the World Health Organization (65).

The dependent variable (SP18) covers the percentage of respondents aged 55–64 (men and women separately) who assess their health as “good” or “very good.”

### Explanatory Variables

Explanatory variables are selected key indicators of the labor market (KILM) (Table 1), covering the different unemployment aspects. These are labor market participation (KILM1), part-time employment (KILM6), long-term unemployment (KILM11), time-related underemployment (KILM 12), and inactivity rate (KILM13) (Table 1).

**Table 1:** Explanatory variables—selected indicators of the labor market.

	**Variable**	**Description**
1	Labor force participation rate (KILM 1)	A measure of the proportion of a country's working-age population that engages actively in the labor market, either by working or by looking for work
2	Part-time employment as a percentage of total employment (KILM 6)	A measure of employment in the informal economy as a percentage of total non-agricultural work
3	Long-term unemployment (KILM 11)	The unemployment lasting 12 months or more as a percentage of the total unemployment
4	Time-related underemployment (KILM 12)	The time-related underemployment as a percentage of total employment
5	The inactivity rate (KILM 13)	The percentage of the population that is neither working nor seeking work
6	Retirement age (RA-55)	Difference between retirement age and age 55
7	Household income (INCOME)	Mean income by household type—EU-SILC and ECHP surveys, single person household. Household income refers to the total amount of gross revenue generated by the individuals living within one particular household

There are also additional variables that go beyond the characteristics of the labor market. Since the test sample involves persons of pre-retirement age, the variable RA-55 describes the timespan to the retirement for persons aged 55 years (respectively, for women and men), taking into account different retirement schemes in the individual countries. We assume that the proximity of retirement benefits may affect individuals' decisions to stay active or transit into a state of inactivity. A lower value of RA-55 should contribute to higher ratios of inactivity.

The variable INCOME helps to control the impact of the financial situation on health. The level of generated income may also impact the decision to leave the labor market (Table 1).

## Results

### Statistical Analysis

Tables 2–7 present the descriptive statistics for KILM 1, KILM 6, KILM 11, KILM 12, KILM 13, and SP18. Labor force participation (KILM 1) is one of the primary measures for assessing the labor market from the perspective of social conditions, including health. The value of KLIM 1 was, in 2018, the lowest (36.1%) in Romania and the highest in Estonia (74.4%) (Table 2).

**Table 2:** KILM 1—descriptive statistics for the years 2008 and 2018.

	**Women**		**Men**	
	**2008**	**2018**	**2008**	**2018**
Average value	39.4	56.67	57.98	68.27
Average value/maximum value (%)	64.17	76.16	85.77	89.59
Kurtosis	1.11	−1.29	0.12	−1.42
Gini coefficient	0.19	0.13	0.08	0.05
Skewness	0.44	−0.20	−0.99	−0.20
Standard dev.	13.38	12.99	8.82	5.92
Volatility (%)	34	23	15	9

**Table 3:** KILM 6—descriptive statistics for the years 2008 and 2018.

	**Women**		**Men**	
	**2008**	**2018**	**2008**	**2018**
Average value	15.14	15.93	6.91	7.92
Average value/maximum value (%)	51.76	51.40	62.21	61.89
Kurtosis	2.87	3.63	−0.75	–0.85
Gini coefficient	0.20	0.19	0.21	0.21
Skewness	1.24	1.65	−0.07	0.47
Standard dev.	6.08	6.16	2.58	2.94
Volatility (%)	40	39	37	37

**Table 4:** KILM 11—descriptive statistics for the years 2008 and 2018.

	**Women**		**Men**	
	**2008**	**2018**	**2008**	**2018**
Average	56.73	47.87	52.48	52.93
Average value/maximum value (%)	71.72	61.85	64.08	72.12
Kurtosis	2.04	2.68	1.02	−1.12
Gini coefficient	0.09	0.13	0.15	0.14
Skewness	0.90	1.66	0.48	0.06
Standard dev.	10.43	2.98	14.19	12.84
Volatility (%)	18	27	27	24

**Table 5:** KILM 12—descriptive statistics for the years 2008 and 2018.

	**Women**		**Men**	
	**2008**	**2018**	**2008**	**2018**
Average	4.01	4.31	3.67	5.07
Average value/maximum value (%)	28.25	27.11	22.22	30.90
Kurtosis	6.28	7.78	4.98	0.94
Gini coefficient	0.42	0.40	0.61	0.53
Skewness	2.41	2.71	2.23	1.47
Standard dev.	3.82	4.20	4.98	5.34
Volatility (%)	95	98	136	105

**Table 6:** KILM 13—descriptive statistics for the years 2008 and 2018.

	**Women**		**Men**	
	**2008**	**2018**	**2008**	**2018**
Average	60.60	43.33	42.02	31.73
Average value/maximum value (%)	77.10	67.81	70.39	77.59
Kurtosis	−1.11	−1.29	0.12	−1.42
Gini coefficient	0.12	0.17	0.11	0.11
Skewness	−0.44	0.20	0.99	0.20
Standard dev.	13.38	12.99	8.82	5.92
Volatility (%)	22	30	21	19

**Table 7:** Retirement age in analyzed countries (in 2008 and 2018).

	**Women**		**Men**	
	**2008**	**2018**	**2008**	**2018**
Bulgaria	60	61.17	60	61.17
Czechia	59.33	62.67	59.33	62.67
Estonia	60.5	63.25	60.5	63.25
Hungary	61	63.25	61	63.25
Latvia	61.5	63.25	61.5	63.25
Lithuania	60	62.33	60	62.33
Poland	60	60	60	60
Romania	58.42	60.92	58.42	60.92
Slovakia	62	62.42	62	62.42

A decreasing KILM1 disparity over time, measured by the mean share in the maximum value, can also be observed. A smaller disparity applies to the male population than to women (in 2018, 89.59% comparing to 76.16%). The concentration of KILM1 (Gini coefficient) is also minimal, which means an even distribution, especially for men. For women, we also see more significant variability between countries −23% compared to 9% for men (Table 2).

The more significant disparity of KILM6, measured by the average at the maximum value, affects men rather than women. The share of the average maximum in 2018 is 61.89% for men. The Gini coefficient for men and women adopts similar values does not exceed 0.21. Right-hand asymmetry (1.24) for women means that part-time work as a percentage of women's total employment is below average in most countries. For men, we observe a moderate left side asymmetry (0.47) (Table 3).

About half of unemployed people were unemployed for more than 12 months. The long-term unemployment rate in 2008 for women was the lowest in Hungary (43.4%) and the highest (79.1%) in Slovakia. In 2018 KILM11 was the lowest for Poland, compared with the other surveyed countries −33.9%. The KILM11 disparity, measured by the mean's share in the maximum, is similar for men and women. The concentration measured with the Gini coefficient is low both for the men and women population. Distributions are characterized by right-sided strong asymmetry in women's cases (Table 4).

KILM 12 describes the problem of underemployment as a percentage of workers who would like to extend the number of working hours. In 2008 the lowest value of KILM 12 for women was reported in Estonia (1%) and the highest in Poland (14.2%). On average, 4.31% of women and 5.07% of men would like to work longer in 2018. The KILM12 disparity, measured by the share of mean in the maximum value, is high for both women and men. The concentration is high, especially for men—an uneven distribution is observed. Right-side asymmetry is extreme for both sexes, and values are characterized by very high volatility. In Poland, 15.9% of employed women and 16.4% of working men would like to extend their working hours. These values stand out from other countries (Table 5).

The inactivity rate is the proportion of the working-age population excluded from the labor force. The KILM13 rate for women was the lowest in 2008 in Estonia (38.6%) and the highest in Poland −78.6%. In the period 2008–2018, there is a clear positive tendency for female and male populations. In the case of women, the inactivity rate dropped from 60.6 to 43.33% during the years 2008–2018. The lower disparity, measured by the share of mean in the maximum value, applies to men. Men's and women's concentration coefficients are low with weak right-side asymmetry for both subpopulations (2018) (Table 6).

To summarize, the preliminary analysis of variables indicates a vital gender gap. 43.3% of women aged 55–64 are inactive—women do not look for employment for various reasons. It is significantly higher than for men (31.73%). Simultaneously, the number of inactive women decreased substantially between 2008 and 2018 (from 60.6 to 43.33). The reasons may be related to reforms in the area of retirement policy aiming to increase the retirement age, especially for women, and equalize retirement age for both sexes (Table 6). This change had a significant positive impact on the activity of this age group increasing participation rates—from 39.4 to 56.67 for women and from 57.98 to 68.27 for men. However, in many CEE countries, women still retire earlier than men, which usually means passing into a state of inactivity.

About half of unemployed people are unemployed for more than 12 months, both women and men. Only a tiny proportion of those who work (4.31% of women and 5.07% of men) would like to do more, although the situation is sharply different between countries. This need for extra hours of work is evident in Poland and Romania.

The variable SP18 describes the percentage of persons who assess their health as “*good*” and “*very good*.” We observe that this percentage for women significantly increases between 2008 and 2018, while for men slightly decreases. The disparity of the variable SP18 is high both for men and women. A moderate left side asymmetry similar for both men and women can be observed.

Some analyzed variables show very high volatility or inequality between countries. Hence, in the next step, we test the significance of differences between means of selected variables for women (Table 8) and men (Table 9) subpopulations. The vast majority of differences between means are statistically significant (statistically significant differences are marked as gray areas). In particular, there are no significant differences for Latvia (LV).

**Table 8:** SP18—descriptive statistics for the years 2008 and 2018.

	**Women**		**Men**	
	**2008**	**2018**	**2008**	**2018**
Average value	27.06	40.51	45.89	45.58
Average value/maximum value (%)	75.38	73.39	76.74	72.69
Kurtosis	−0.72	−0.70	−0.83	0.05
Gini coefficient	0.13	0.13	0.10	0.13
Skewness	−0.59	−0.26	0.48	–0.46
Standard dev.	6.82	9.54	8.48	10.70
Volatility (%)	25	24	18	23

**Table 9:** Test for significance of mean differences for women (statistically significant differences market as gray areas).

**Variable**	**Country**	**LV**	**HUN**	**PL**	**LIT**	**EST**	**SL**	**CR**	**ROM**	**BUL**
KILM11	LV	56.771	47.718	49.979	54.200	52.578	74.736	48.993	–	63.500
	HUN		8.373	6.318	2.521	3.722	−19.131	7.576	–	−7.480
	PL			−1.947	−5.839	−4.021	−26.078	−1.142	–	−15.776
	LIT				−3.823	−2.161	−24.044	0.888	–	−13.606
	EST					1.405	−21.127	4.927	–	−9.958
	SL						−20.457	3.091	–	−10.411
	CR							26.299	–	13.303
	ROM								–	−15.416
	BUL									–
KILM12	LV	4.957	4.544	19.339	3.200	1.254	2.032	2.071	3.651	4.545
	HUN		0.899	−17.992	4.289	9.546	6.869	7.332	2.895	0.770
	PL			−18.518	3.287	8.498	5.909	6.295	1.982	−0.001
	LIT				20.922	23.793	22.185	22.631	19.754	17.502
	EST					5.961	3.151	3.388	−1.130	−2.726
	SL						−2.246	−2.672	−6.357	−6.921
	CR							−0.111	−3.893	−4.957
	ROM								−4.126	−5.152
	BUL									−1.691
KILM13	LV	40.379	65.350	70.071	43.593	34.364	63.871	57.264	65.771	54.286
	HUN		−25.666	−29.408	−3.112	6.735	−20.486	−16.524	−35.723	−13.295
	PL			−4.511	20.351	33.160	1.254	7.640	−0.553	10.228
	LIT				24.014	36.712	5.125	11.726	5.317	14.159
	EST					9.258	−16.494	−12.274	−26.478	−9.412
	SL						−26.481	−23.242	−47.796	−19.717
	CR							5.415	−1.950	7.728
	ROM								−10.326	2.645
	BUL									13.455
SP18	LV	17.907	20.307	29.979	30.371	33.286	34.564	41.064	42.428	44.150
	HUN		−2.851	−13.604	−13.032	−20.101	−15.695	−27.569	−31.122	−25.914
	PL			−10.542	−10.223	−16.229	−13.121	−23.813	−26.924	−22.945
	LIT				−0.384	−3.902	−4.086	−12.105	−14.338	−13.165
	EST					−3.169	−3.560	−10.879	−12.843	−12.150
	SL						−1.243	−9.749	−12.307	−11.108
	CR							−5.990	−7.522	−7.833
	ROM								−1.664	−2.974
	BUL									−1.729

### GMM Model

Separate models for men and women due to the significant variation in variable values are estimated. Models 1 and 2 explain the self-perceived health for women and men, respectively. Both models are estimated using explanatory variables number 1–5 (Tables 10, 11).

**Table 10:** Test for significance of mean differences for men (statistically significant differences market as gray areas).

**Variable**	**Country**	**LV**	**HUN**	**PL**	**LIT**	**EST**	**SL**	**CR**	**ROM**	**BUL**
KILM11	LV	57.907	48.250	54.709	52.893	49.207	74.450	47.393	62.843	49.193
	HUN		3.028	0.814	2.794	2.815	−6.385	4.500	−2.656	2.997
	PL			−1.356	−1.434	−0.233	−7.010	0.240	−4.460	−0.238
	LIT				0.458	1.171	−4.500	1.725	−2.034	1.204
	EST					1.174	−8.136	2.290	−5.131	1.250
	SL						−6.909	0.522	−4.293	0.004
	CR							8.893	4.311	7.215
	ROM								−6.309	−0.543
	BUL									4.551
KILM13	LV	34.9	50.407	33.386	33.493	47.971	41.393	33.871	40.586	46.107
	HUN		−5.651	1.094	0.848	−6.067	−5.692	0.651	−3.344	−9.226
	PL			6.265	5.902	0.766	3.466	5.861	3.398	1.632
	LIT				−0.066	−6.881	−7.457	−0.317	−4.347	−11.043
	EST					−6.273	−5.605	−0.212	−3.751	−8.582
	SL						3.341	6.261	3.159	0.926
	CR							5.717	0.553	−5.590
	ROM								−3.684	−8.864
	BUL									−3.640
SP18	LV	22.821	23.586	30.314	32.114	33.229	38.136	44.507	51.364	52.616
	HUN		−0.503	−6.312	−4.118	−6.419	−5.522	−14.404	−16.386	−18.701
	PL			−4.791	−3.586	−5.398	−5.064	−12.438	−14.647	−16.486
	LIT				−0.825	−1.924	−2.884	−10.212	−12.813	−15.025
	EST					−0.456	−1.812	−5.230	−7.620	−8.450
	SL						−1.676	−6.354	−9.161	−10.479
	CR							−2.223	−4.414	−4.971
	ROM								−3.636	−4.635
	BUL									−0.640

**Table 11:** GMM model 1, dependent variable—SP18(f).

**Variable**	**Coefficient**	**Standard error**	***t*-ratio**	***p*-value**
Const	87.6358^***^	2.58565	33.89	<0.0001
KILM11	−0.0540644^*^	0.0298267	−1.813	0.0699
KILM12	−0.0742690^**^	0.0344980	−2.153	0.0313
KILM13	−0.764447^***^	0.0358556	−21.32	<0.0001
Latvia	−35.4239^***^	1.20294	−29.45	<0.0001
Hungary	−14.4547^***^	1.09374	−13.22	<0.0001
Lithuania	−20.7720^***^	1.33958	−15.51	<0.0001
Estonia	−25.1447^***^	1.19975	−20.96	<0.0001
Bulgaria	1.78340	1.16948	1.525	0.1273

* *significance level α = 0.1*,

** *significance level α = 0.05, and*

*** *significance level α = 0.01*.

The general form of the model 1and 2 are as follow:

$$\begin{array}{lll} \text{SP}18 & = & a_{0}+a_{1}KILM1+a_{2}KILM6+a_{3}KILM11+a_{4}KILM12\\ \;+a_{5}KILM13+\beta_{1}\text{BUL}+\beta_{2}\text{CR }+\beta_{3}\text{EST }+\beta_{4}\text{LV }\\ \;+\beta_{5}\text{LT }+\beta_{6}\text{HUN}+\beta_{7}\text{PL}+\beta_{8}\text{ROM}+\beta_{9}\text{SL}+\varepsilon_{i} \end{array}$$

The models do not contain any structural changes. The Chow test, in both cases, confirms the stability of the parameters. The null hypothesis assumes the absence of structural changes. For the male model, *F*_(5, 97)_ = 0.59331, with *p-*value 0.7051, and for women, *F*_(3, 113)_ = 2.48539 with *p*-value 0.0643.

In model 1, estimated for women aged 55–64, three variables explain the self-perceived health—long-term unemployment (KILM11), time-related underemployment (KILM12), and inactivity rate (KILM13). Other variables did not enter the model. For KILM 11, the partial regression coefficient is −0.0540644 with an error of ±0.0298267, which allows the following interpretation: when the long-term unemployment rate increases by 1 (%), health perception decreases by 0.054 (%), provided that the values of other variables do not change. When the time-related underemployment (KILM12) increases by 1%, the self-perceived health decreases by 0.0742690 (%). Analogically, the partial regression coefficient for KILM13 in (%) is −0.764447, with an error of 0.0358556. Hence, if the inactivity rate increases by 1 (%) on average, the perception of health decreases by 0.764447 (%), provided that the size of other variables does not change (Table 11).

The dependent variable's [SP18(f)] arithmetic mean is 31.45357 and the standard deviation of the dependent variable is 10.89073. GMM criterion: Q = 1.69439e-022 (TQ = 1.89772e-020).

The self-perceived health of men aged 55–64 (model 2) is explained by only two variables: long-term unemployment (KILM11) and inactivity rate (KILM13).

The partial regression coefficient for KILM 11 is −0.112940 with an error of ±0.0422647, which allows the following interpretation: as the long-term unemployment rate increases by 1 (%), health perception decreases by 0.112 (%), provided that the values of other variables do not change. Analogically, the partial regression coefficient for KILM13 in (%) is −0.608790, with the error of 0.0722067. Hence, if the inactivity rate increases by 1 (%) on average, the perception of health decreases by 0.609 (%), provided that the size of other variables does not change (Table 12).

**Table 12:** GMM model 2, dependent variable—SP18 (m).

**Variable**	**Coefficient**	**Standard error**	***z***	***p*-value**
Const	71.1119	3.25845	21.82	<0.0001
KILM11	−0.112940^***^	0.0422647	−2.672	0.0075
KILM13	−0.608790^***^	0.0722067	−8.431	<0.0001
Latvia	−20.5037^***^	1.29170	−15.87	<0.0001
Hungary	−11.3895^***^	2.18675	−5.208	<0.0001
Estonia	−14.2939^***^	1.11234	−12.85	<0.0001
Lithuania	−12.6338^***^	1.61623	−7.817	<0.0001
Poland	−3.12142^**^	1.30629	−2.390	0.0169
Bulgaria	12.0580^***^	1.54434	7.808	<0.0001
Romania	15.1295^***^	1.53300	9.869	<0.0001

*significance level α = 0.1,

** *significance level α = 0.05, and*

*** *significance level α = 0.01*.

Models 3 (for women) and 4 (for men) are estimated using all explanatory variables. It allows for analyzing the potential effect of retirement age and income.

The general form of the model 3 and 4 are as follow:

$$\begin{array}{lll} \text{SP}18 & = & \alpha_{0}+\alpha_{1}KILM1{+\alpha }_{2}KILM6+\alpha_{3}KILM11+\alpha_{4}KILM12\\ \;+\alpha_{5}KILM13{+\;\alpha }_{6}\text{INCOME }{+\;\alpha }_{7}\text{ RA}-55+\beta_{1}\text{BUL}\\ \;+\beta_{2}\text{CR }+\beta_{3}\text{EST }+\beta_{4}\text{LV }+\beta_{5}\text{LT }+\beta_{6}\text{HUN}+\beta_{7}\text{PL}\\ \;+\beta_{8}\text{ROM}+\beta_{9}\text{SL}+\varepsilon_{i} \end{array}$$

The models do not contain any structural changes. The Chow test in both cases confirms the stability of the parameters. The null hypothesis is the absence of structural breaks in a time series. In the male model (model 4), this is a low value [*F*_(4, 111)_ = 7.10122, *p*-value = 3.96467e-005], and for women (model 3), it is indisputable [*F*_(6, 93)_ = 1.38501, *p*-value = 0.228855].

When the additional variables (INCOME, RA-55) are included, the long-term unemployment ratio does not enter the model. The partial regression coefficient for KILM 12 is −0.187317 with an error of ±0.0623989, which allows the following interpretation: as the time-related underemployment rate increases by 1 (%), health perception decreases by 0.187317 (%), provided that the values of other variables do not change. Analogically, the partial regression coefficient for KILM13 is −0.695259, with an error of 0.0544718. Hence, when the inactivity rate increases by 1 (%) on average, the perception of health decreases by 0.695259 (%), provided that other variables do not change (Table 13).

**Table 13:** GMM model 3, dependant variable—SP18(f).

**Variable**	**Coefficient**	**Standard error**	***z***	***p*-value**
Const	89, 3495	4, 49566	19.87	<0.0001
KILM12	−0.187317^***^	0.0623989	−3.002	0.0027
KILM13	−0.695259^***^	0.0544718	−12.76	<0.0001
INCOME	0.00215923^***^	0.000419313	5.149	<0.0001
RA-55	−2.19702^***^	0.467027	−4.704	<0.0001
Latvia	−36.2116^***^	1.22729	−29.51	<0.0001
Hungary	−17.3224^***^	1.84488	−9.389	<0.0001
Poland	−6.51857^***^	2.24979	−2.897	0.0038
Lithuania	−24.5082^***^	1.23516	−19.84	<0.0001
Estonia	−30.2173^***^	1.25956	−23.99	<0.0001
Slovakia	−5.80266^***^	1.86952	−3.104	0.0019
Czechia	−9.90302^***^	1.95995	−5.053	<0.0001

*significance level α = 0.1, **significance level α = 0.05, and

*** *significance level α = 0.01*.

This model also includes the impact of household income and retirement age. When the household income increases by 1 (%), the perceived health very slightly increases by 0.00215923 (with the error of 0.000419313). In the case of RA-55, the strength of the relationship is significantly higher. When RA-55 increases by 1 (%), the perceived health decreases by 2.19702 (with the error of 0.467027). It can be interpreted as follow: women who expect a more extended period of work until retirement assess their health state as worse.

The dependent variable's [SP18(f)] arithmetic mean is 31.45357 and the standard deviation of the dependent variable is 10.89073. GMM criterion: Q = 6.8816e-024 (TQ = 7.70739e-022).

Model 4 also includes additional variables (INCOME, RA-55). In this case, only one variable describing the labor market enters the model (the long-term unemployment ratio). The partial regression coefficient for KILM 11 is −0.0744426 with an error of ±0.0298090, which allows the following interpretation: as the long-term unemployment rate increases by 1 (%), health perception decreases by 0.0744426 (%), provided that the values of other variables do not change (Table 14).

**Table 14:** GMM model 4, dependant variable—SP18(m).

**Variable**	**Coefficient**	**Standard error**	***z***	***p*-value**
Const	29.2000	4.04098	7.226	<0.0001
KILM11	−0.0744426^**^	0.0298090	−2.497	0.0125
INCOME	0.00302122^***^	0.000459543	6.574	<0.0001
RA-55	2.30497^***^	0.489014	4.714	<0.0001
Latvia	−31.3491^***^	1.74868	−17.93	<0.0001
Hungary	−31.0303^***^	2.25511	−13.76	<0.0001
Estonia	−30.4854^***^	2.57825	−11.82	<0.0001
Lithuania	−23.4199^***^	1.63270	−14.34	<0.0001
Poland	−30.7461^***^	1.22612	−25.08	<0.0001
Slovakia	−17.5330^***^	2.81387	−6.231	<0.0001
Czechia	−17.3291^***^	2.58550	−6.702	<0.0001

*significance level α = 0.1,

** *significance level α = 0.05, and*

*** *significance level α = 0.01*.

For women, the household income impacts self-perceived health marginally. When the household income increases by 1 (%), the perceived health very slightly increases by 0.00302122 (with the error of 0.000459543). In the case of RA-55, a healthy relationship but in a different direction is observed. When RA-55 increases by 1 (%), the perceived health increases by 2.30497% (with the error of 0.489014). Hence, men who expect to work for a longer time assess their health state as better.

The dependent variable's [SP18(f)] arithmetic mean is 31.45357 and the standard deviation of the dependent variable is 10.89073. GMM criterion: Q = 6.8816e-024 (TQ = 7.70739e-022).

To summarize, the labor market characteristics which influence the perceived health are, depending on the model, long-term unemployment (KILM 11), time-related underemployment (KILM 12), and inactivity rate (KILM 13). All those factors affect perceived health negatively. When we include income, we observe a positive relationship; however, it is relatively feeble. It seems that the retirement age plays an important role. Estimated models suggest that women who expect a longer timespan of work perceive their health as worse. This relationship is relatively high and statistically significant. For men, and the opposite relationship is detected—men who expect to work up to longer age assess their health as better. It is potentially an exciting finding but, in our opinion, requires further research.

## Discussion

The research involves people aged 55–64. It is a period in life when health progressively deteriorates. Lack of success in the labor market, like losing a job, can also contribute to the deterioration of health. Previous studies do not expressly answer the link between employment and health.

As summarized in section Literature Context, several studies confirm the detrimental impact of unemployment on health outcomes, regardless of the dependent variables. The negative effect of being unemployed is reflected in higher mortality and morbidity rates or lower health state self-assessment (13, 17, 22–24, 29, 44, 66–68). This relationship is perceived as being bidirectional. Hence the willingness to seek a job is also influenced by the health state (49). Lower self-assessed health may result from depression symptoms that often accompany unemployment or inactivity (69). According to Krug and Eberl, workers, who enter unemployment with lower health, assess their health as worse (29).

The impact of unemployment on health is evident in various health indicators—the authors usually analyze health in the context of mortality, maturity, and self-health (SAH). If mortality is examined, most studies, especially earlier ones, suggest a negative impact. In one of earlier studies, Brenner, based on British data, concludes that a negative impact of unemployment is expressed by slowing the decline in mortality (11). Also, Wilson and Walke demonstrable an adverse effect on health by increased mortality experience of Britain unemployed (8). Morris and colleagues estimate that unemployed middle-aged British men were twice as likely to die in the following 5.5 years as those who remained continuously employed (70). Similar dependencies confirm, in Finland, Martikainen and Valkonen (71), Tapia Granados in Spain (72), Crost and Friedson in the USA (23), in Sweden: Eliason and Storrie in Sweden (14) and Garcy and Vågerö (19). Ariizumi and Schirle confirm, using Canadian data, that a one p.p. increase in the unemployment rate lowers the predicted mortality rate by nearly 2%. Zagozdzon et al. find that the Polish unemployed are at greater risk of death than the overall population (73).

Panel studies in this area are not so consistent. Based on data from 13 European Union countries, Economou et al. confirm a strong, positive relationship between adverse economic conditions, including unemployment and mortality (22). On the other hand, Tapia Granados et al. find, using data from 27 European countries with over one million citizens, that an increase of one percentage point in the unemployment rate is associated with a reduction of 0.5% in the rate of age-adjusted mortality (38). Gerdtham and Ruhm, using a fix-effect panel with 23 OECD countries, including countries of CEE, confirm that one p.p. decrease in the unemployment rate is associated with the growth of 0.4% in total mortality (33).

When morbidities describe a health state, results are, by far, more consistent. They usually indicate a higher risk of heart disease (70, 74–81). Individual studies conducted in different countries, including CEE countries, examine this adverse effect in more detail and identify various causes of increased risk, but are most often associated with stress associated with job loss, lifestyle changes, and different physical activity patterns.

From the point of view of this research, the most interesting are the studies in which respondents assess their health independently (SAH). Binder and Coad conclude that unemployment cause a substantial decrease in subjective well-being in the UK population (16). László et al., using data from 3 population-based studies (16 countries including CEE countries), find that job insecurity was significantly associated with an increased risk of poor health in the Czech Republic, Denmark, Germany, Greece, Hungary, Israel, the Netherlands, Poland, and Russia. In contrast, this relationship is statistically insignificant in other countries analyzed countries (82). Based on Spanish data, Urbanos-Garrido and Lopez-Valcarcel confirm the detrimental impact on unemployment on SAH.

By contrast to that, Tøge and Blekesaune, using data from 28 European Countries, including CEE ones, suggest that unemployment and health are partly due to decreased self-rated health as people enter unemployment. This study also emphasizes the detrimental impact on older workers' health (83). Against this background, Krug and Eberl get exciting results. Based on data for Germany, they find exciting time-lags related to the relationship between unemployment and SAH. During the first year of unemployment, SAH is significantly lower than 2 or more years before entering unemployment and when the period of unemployment is longer than 1 year (29).

In light of the presented findings, only long-term unemployment impacts health detrimentally. The constant lack of professional activity has a similar influence. Additionally, women's self-assessed health suffers from time-related underemployment. Previous studies show that women are at higher risk of underemployment as underemployment is most common in women's professions (84).

This study also confirms that among the group of elderly workers, the perceived state of health is affected not simply by unemployment but primarily by its structure. Short-term unemployment is often perceived as desirable—it allows job seekers to find suitable employment. It can be a time of relief from a daily routine for stressed workers, especially when social support schemes offer satisfying financial security levels. However, in light of those findings, long-term unemployment harms self-assessment health. However, this effect is more substantial for men. This negative impact is possibly rooted not only in lower economic status but social factors that may also contribute to lower perceived health status (26). Those results contrast with the findings of Kostrzewski and Worach-Kardas, who investigated a group of 454 Polish unemployed aged 45 years and older—they conclude that a period of unemployment did not significantly contribute to the self-rated health (85).

In contrast to many earlier studies, significant gender differences are not observed, which is a little bit puzzling in the context of research suggesting that unemployment has a more substantial impact on men's health (16, 44, 86–88). For example, Eliason and Storrie find that the overall men's mortality is 44% higher, while there is no impact on female mortality (14).

Women are also at higher risk of underemployment due to the structure of jobs they usually take (84). To a more significant extent, women are permanently excluded from longer working-hour (89). According to obtained results, this need for extra working hours harms their health assessment. It is in line with previous studies. Friedland and Price found that underemployed workers report lower health and well-being (90).

Apart from the problem of unemployment, presented findings highlight the importance of labor market inactivity. Three of four estimated models suggest the negative impact of inactivity on health—this impact is more substantial compared to other characteristics of the labor market. At the age of about 60 years, both women and men usually decide when to retire. From the labor market perspective, it means a transition into a state of inactivity or significantly reduced working hours. According to Eurostat, 15.9% of unemployed aged 55–64 who left the labor market chose early retirement, while 15.8% were forced to retire due to illness or disability (91). It indicates the bidirectional relationship between inactivity and health; as French reports, unhealthy people retire earlier (92). Also, Disney concludes that poor health is a predictor of individual retirement behavior among workers aged 50 until state pension age (93). Men in poor health are expected to retire 1–2 years earlier—this effect is visible after correcting potential endogeneity of self-rated health problems (94).

The lack of activity, rather than unemployment, might be a source of health state deprivation. Inactivity has a significant negative impact on both sexes' perceived state of health (95). Hence, social and economic consequences of inaction are often related to retirement and adversely affect well-being (96), especially in a group of older employees. This detrimental impact is reported in earlier studies—Behncke concludes that retirement significantly increases the risk of being diagnosed with a chronic condition, increased risk factors, or physical activity problems (97). Retirement may also be related to a decrease in cognitive skills (98). According to Dave, retirement cause an increase in difficulties associated with mobility, daily activities, illness conditions, and a decline in mental health (99).

There is a group of researchers that reports a positive impact of retirement on health. Che concludes that the probability of “fair” or “poor” self-reported health among white-collar workers decreases substantially after retirement (100, 101). Some studies suggest that retirement improves subjective health status and mental health due to lower stress and a better lifestyle (102, 103). While researchers often report a positive impact on mental health, at the same time, perceived general health, and physical health may suffer (104, 105). It seems that for better-educated workers, the decision to retire is more beneficial (100, 104, 106, 106).

The results also suggest that the retirement age influences importantly perceived health, but this relationship's direction is damaging for women and positive for men. It contrasts with studies suggesting that the statutory retirement age is unrelated to an individual's health (101). Although the study confirms that the retirement age affects perceived health, the differences between women and men require further research.

## Limitations of the Study

As previously mentioned, CEE countries form a homogenous group in terms of economy, demography, and social systems, including retirement schemes, making the estimation results more reliable. The research results generally confirm the relationship revealed in previous studies, both on the direction and strength of the dependencies. However, there are a few limitations to consider. Firstly, the previous findings are significantly inconsistent, so it is difficult to identify some universal conclusions.

Secondly, most studies in this area cover data from highly developed countries—the US and Western Europe. Research in developing countries is relatively scarce and most often points to a positive link between unemployment and health (68, 107, 108). However, comparisons with developing countries do not appear to be justified, as there are characterized by very restricted social policy, which plays an essential role in the consequences of unemployment. Moreover, while there are still some development differences between eastern and western Europe, CEE countries are very close to Western European neighbors when it comes to social security systems. Unfortunately, previous studies on CEE countries usually cover individual populations (Poland, Czech Republic) and are usually based on mortality rates. In panel studies, CEE countries appear as part of a larger research sample covering European or OECD countries.

Therefore, it is justified to conduct further comparative studies to capture possible differences between Western Europe countries and CEE countries. Although the microeconomic structure of unemployment in the nations of eastern Europe appears to be similar to the industrialized west (109)—some studies suggest that populations of CEE can be more sensitive to business cycle fluctuations, independent of gender (110). At the same time, Bambra and Eikemo report the minor relative inequalities between employed and unemployed in the Southern and Eastern welfare states (111). That suggests the potential direction of further research.

## Conclusions

The process of demographic change is an undeniable fact. In the coming years, pre-retirement age employees will form a significant group in the labor market. This part of the population is, by nature, at higher risk of health state deterioration, as a part of the aging process. Any difficulties in the labor market may boost these problems and push workers out of the labor market, as we remember the bidirectional relationship between health and its socio-economic determinants. Taking into account the potential shortage of workers, it may pose severe problems for the economy.

Unemployment, especially when it has a long-term character, harms the health of employers from the older age groups—unemployed assess their health state as worse than those who have a job. Interestingly, the impact of labor inactivity is, importantly, more wasting than an unemployed status. Although this effect's mechanism requires further studies, we presume that permanent resignation from job-seeking reinforces the social gradient's detrimental impact, leading to a worsen economic situation or deepen social isolation.

Apart from long-term unemployment, women also suffer from time-related underemployment. It suggests that public politics promoting employment should concentrate not only on unemployed but also part-time workers.

Being inactive is a natural consequence of taking retirement. In this context, the problem of retirement age plays an important role. All CEE countries, except Poland, gradually increase the statutory retirement age, especially for women. It can be a source of, on average, lower perceived health status (compared to men).

To conclude, there are several advantages of an active senior policy aimed at galvanizing older workers' activities, promoting employment, and gaining new competencies. This tailor-made policy should address the identified determinants of labor inactivity to prevent early retirement.

This study has several advantages comparing to the existing literature: firstly, to our best knowledge, this is the first study on this group of countries, which may open the discussion on potential differences between European countries. Secondly, the analysis covers diverse aspects of unemployment (overall unemployment, long-term unemployment, underemployment, and inactivity), catching the most critical dependencies. Thirdly, the research includes the variable describing the proximity of retirement to control potential differences of retirement schemes, which plays a crucial role in studies on older workers.

## Data Availability Statement

Publicly available datasets were analyzed in this study. This data can be found here: https://ilostat.ilo.org/.

## Author Contributions

PU-J and AB: conceptualization and writing—original draft preparation. PU-J: data curation, formal analysis, methodology, resources, and visualization. AB: investigation and writing—review and editing. All authors contributed to the article and approved the submitted version.

## Conflict of Interest

The authors declare that the research was conducted in the absence of any commercial or financial relationships that could be construed as a potential conflict of interest.
